# Nutritional status and physical function of older adults living in sub-Saharan Africa: a systematic review

**DOI:** 10.1186/s40795-026-01351-y

**Published:** 2026-05-11

**Authors:** Onosolesena D. Idiakheua, James Odhiambo Oguta, Elvis O. Wambiya, Elizabeth A. Williams, Viren Ranawana

**Affiliations:** 1 Division of Population Health, School of Medicine and Population Health, University of Sheffield, Sheffield, S1 4DA UK; 2 Department of Biochemistry, Faculty of Life Sciences, Ambrose Alli University, Ekpoma, Edo State Nigeria; 3 Division of Clinical Medicine, School of Medicine and Population Health, University of Sheffield, Sheffield, S10 2RX UK

**Keywords:** Nutrition status, Physical function, Older persons, Sub-Saharan Africa

## Abstract

**Background:**

Adequate nutrition is a cornerstone of healthy ageing, yet malnutrition is an escalating concern among older adults in sub-Saharan Africa (SSA). Despite its impact on independence and chronic disease risk, empirical syntheses of the relationship between nutritional status and physical function in this region remain scarce.

**Objective:**

To systematically review the prevalence of malnutrition among older adults in sub-Saharan Africa and its relationship with physical function.

**Methods:**

A systematic search was conducted across five electronic databases for peer-reviewed studies published in English between 2000 and 2025. Study selection, data extraction, and quality assessment were performed independently by two reviewers. The review targeted studies that assessed nutritional status (e.g., body mass index, Mini Nutritional Assessment) and physical function (e.g., activities of daily living, handgrip strength) in SSA populations aged 60+. Narrative synthesis was used to synthesise and report data outcomes.

**Results:**

Fifteen studies met the inclusion criteria. The prevalence of undernutrition (malnutrition) varied widely, ranging from 1.7% to 75%. Functional impairment was significant: dependency in activities of daily living (ADL) ranged from 9% to 76.5%, while instrumental ADL dependency ranged from 29% to 72.7%. Notably, low to moderate handgrip strength (< 16 kg; ≤27 kg) was also reported.

**Conclusion:**

The included studies reported associations between nutritional status and physical function among older adults in SSA. However, the predominantly cross-sectional nature of the included studies precludes establishing a causal relationship. Furthermore, the high variation in prevalence observed across studies may arise from true population differences and methodological differences. A geographical bias toward urban settings was identified, underscoring the need for longitudinal research and data from rural populations to inform targeted healthy ageing interventions.

PROSPERO registration CRD42023430056.

**Supplementary Information:**

The online version contains supplementary material available at 10.1186/s40795-026-01351-y.

## Introduction

The global demographic landscape is shifting toward an ageing population, a trend evident in sub-Saharan Africa (SSA) as well. In 2021, individuals aged 65 and older accounted for 6.9% of the region’s population, a figure projected to nearly double to 11.1% by 2050 [1]. This rapid demographic transition necessitates a comprehensive understanding of the health and nutritional challenges unique to older adults in this socio-economic context.

Malnutrition, a term that refers to a lack (undernutrition) or excess (overnutrition) of nutrient intake [2], represents a significant public health burden for older adults, with global prevalence estimates ranging from 23% to 46% [3]. The word malnutrition is used interchangeably with undernutrition, a term that encompasses underweight, which arises from insufficient food intake, impaired food absorption, or poor biological utilisation of nutrients [4]. The severity of this issue is amplified in low- and middle-income countries (LMICs) by pervasive poverty and limited dietary diversity [5–7]. In SSA specifically, epidemiological data indicate a malnutrition incidence of 5–30% among community-dwelling older adults, rising sharply to 20–60% in hospital settings [8]. While adequate nutrition is a cornerstone of recovery from illness and infection, achieving it remains a challenge for older Africans who face physiological decline, limited social support, and reduced mobility [9–11].

Ageing is characterised by a progressive decline in physiological systems, often exacerbated by income loss and restricted access to healthcare [12–15]. Central to this decline is the erosion of physical function—the ability of an individual to perform daily activities and tasks that require physical strength [16–18]. Several tools are available for measuring physical function in older adults, including handgrip strength used in measuring muscle strength to assess the amount of force a muscle can produce with a single maximal effort [16], gait speed used to measure physical performance to assess whole body function related with mobility [16], Short Physical Performance Battery (SPPB) test assesses lower extremity functional performance using timed actions of standing balance, gait speed and lower extremity strength [19], Timed Up and Go test a physical performance measure predominantly used to evaluate walking and active stability [20], activities of daily living (ADLs), and instrumental activities of daily living (IADLs).

**Fig. 1:**
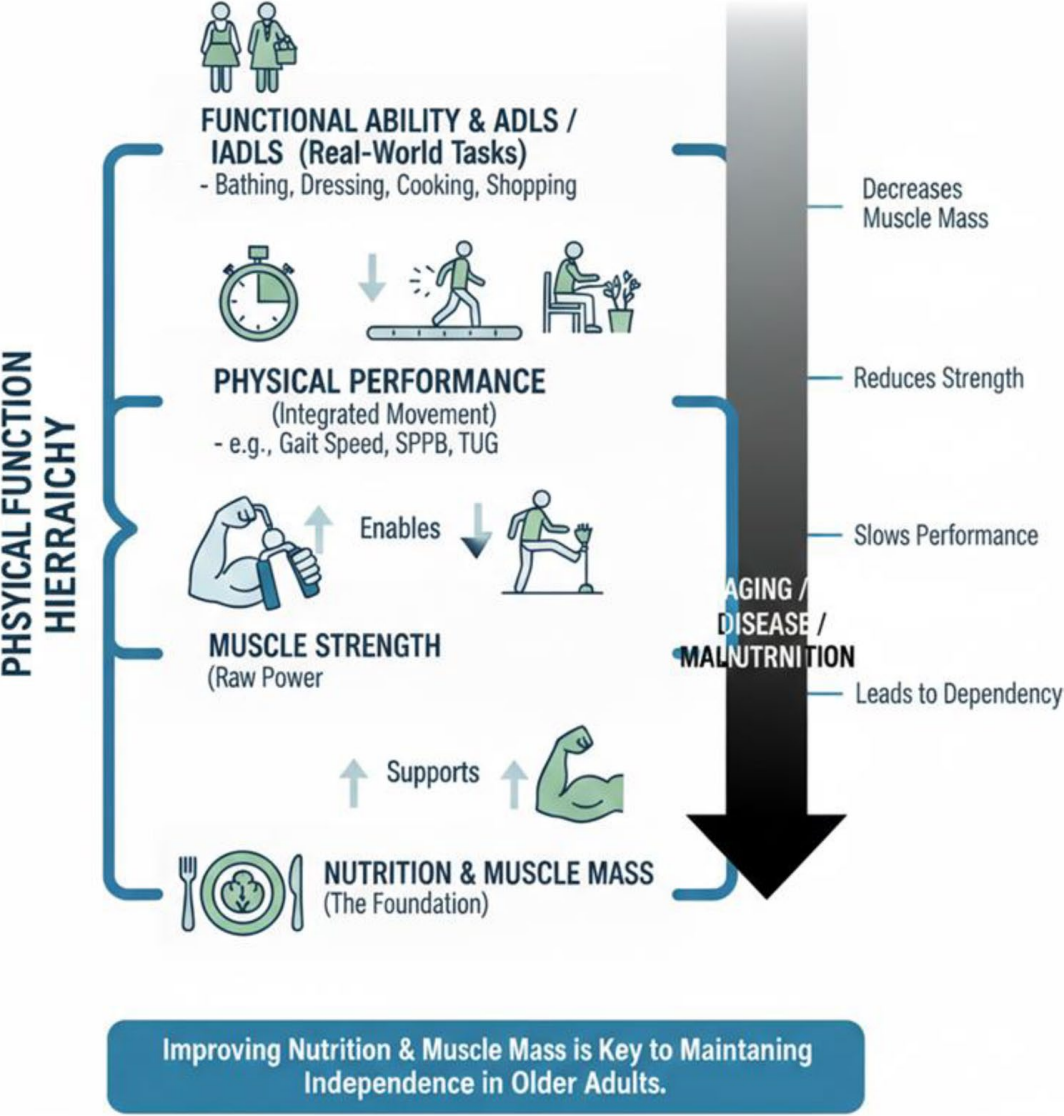
Diagram showing the interrelationship between nutrition and physical function, adapted from Fried et al. [33]. Abbreviations: ADL- activities of daily living; IADL- instrumental activities of daily living; SPPB- short physical performance battery; TUG- timed up & go

Malnutrition and physical function are inextricably linked (Fig. 1): age-related transformations such as anorexia of ageing, malabsorption, and sarcopenia create a feedback loop that leads to frailty, immobility, and increased dependency [21, 22]. Physical function is significantly linked to nutritional well-being in older adults [23, 24]. Physical limitations often create barriers to achieving optimal nutrition, as mobility difficulties and difficulty lifting utensils can negatively impact older adults’ access to food from shops and preparation [23, 25]. Nutritional status and physical function are two critical areas that significantly affect the health trajectories and health-related quality of life of older adults [26]. The state of nutrition in older adults is crucial for bolstering immune health [27] preserving muscle and bone integrity, and averting chronic illnesses [28, 29]. Findings indicate that nutritional status and physical function are significant, interrelated elements that affect health in older adults [30, 31]. Undernutrition has the potential to hasten functional decline, while optimal nutrition can improve physical capacity and provide a protective effect against functional losses. With the ageing of global populations, it is becoming ever more essential to comprehend the intricate connections between nutritional status and physical function, as well as their links to health and well-being [32].

The Global Leadership Initiative on Malnutrition (GLIM) has provided standardised criteria for screening and assessing undernutrition, emphasising the need to understand these linkages in at-risk populations [34]. To date, systematic reviews in SSA on the older adult population have largely focused on the socio-economic determinants of nutritional status or dietary diversity [35–38].

Despite the rising prevalence of the ageing population in sub-Saharan Africa, the geriatric healthcare infrastructure remains critically underdeveloped. While individual studies have documented high rates of both undernutrition and functional impairment, there is a lack of synthesised evidence characterising the interaction between these two conditions. In the absence of a clear understanding of how nutritional deficits drive physical dependency—and vice versa—within the African socio-economic context, public health interventions remain fragmented. Without a systematic synthesis to identify the strength of these associations and the specific tools used to measure them, it is impossible to develop targeted, evidence-based strategies to mitigate the looming crisis of frailty and dependency in the region. Therefore, this systematic review aims to synthesise the current evidence on the nutritional status and physical function of older adults residing in sub-Saharan Africa.

### Research question

Is nutritional status associated with physical function in older adults living in sub-Saharan Africa?

### Objectives

The specific objectives are [1] to systematically review the literature on the prevalence of nutritional status of older adults living in sub-Saharan Africa and [2] assess its relationship with the physical function of older adults.

## Methodology

This systematic review was conducted in accordance with the Preferred Reporting Items for Systematic Reviews and Meta-Analysis (PRISMA) guidelines [39]. The review was registered in the International Prospective Register of Systematic Reviews (PROSPERO registration number CRD42023430056).

### Search strategy

A single reviewer (O.D.I) independently performed the initial searches, with a revised search conducted by two reviewers (O.D.I & E.O.W). A pilot search was conducted on the Medline Electronic database to test the search strategy and obtain preliminary studies on the review topic. Comprehensive searches were conducted on Medline, Embase, Web of Science, Africa Index Medicus (AIM) and African Journals Online (AJOL). Hand searches were performed on Google Scholar, citation follow-up was conducted, and the reference lists of included studies were reviewed for additional references and citations. The PICOS (Table 1) framework for systematic reviews was used to structure the searches’ key free search terms such as “older adults, “nutritional status”, “physical function” and “sub–Saharan Africa”, alongside medical subject heading (MeSH) terms, for example, “micronutrient status”, to capture primary studies examining nutritional status and physical function in micronutrient studies. The search terms were combined using the Boolean logic “AND” and “OR”. The searches were limited to English and restricted to articles published from January 2000 to 2023, as the aim of the study was to identify contemporary trends and correlations concerning malnutrition and physical function in older adults (see full search strategy in the supplementary file). A revised search was conducted in January 2026 to capture articles, including those published very recently from 2000 to December 2025.

**Table 1:** Eligibility of studies determination following the PICOS strategy of inclusion and exclusion criteria

Parameter	Inclusion	Exclusion
Population	Adults 60 years and above, both sexes.	Infants, children, adolescents, middle-aged, and adults below 60 years old
Interest	Nutritional Status and Physical Function.	
Comparison	Not applicable	
Outcome	Anthropometric outcomes (BMI), MNA, and physical function (muscle strength, functional mobility, ADL and IADL).	Studies whose outcomes are not nutritional status and physical function. Also, studies with disease-related outcomes
Study types	Observational designs - cross-sectional studies, longitudinal studies, cohorts, experimental designs, RCTs.	Case-control, literature review, systematic reviews, editorials, conference abstracts.

*Abbreviations*: *BMI *body mass index, *MNA *mini nutritional assessment, *ADL *activities of daily living, *IADL *instrumental activities of daily living, *RCTs *randomised control trials

### Eligibility criteria

Studies were eligible for inclusion if they were conducted among older adults aged 60 years and above (the selection of this age is predicated on the region’s life expectancy) in sub-Saharan Africa and evaluated both nutritional status and physical function in a study. Studies were excluded if they were disease-related malnutrition and did not meet the criteria stated above (see Table 1).

### Study selection

**Fig. 2:**
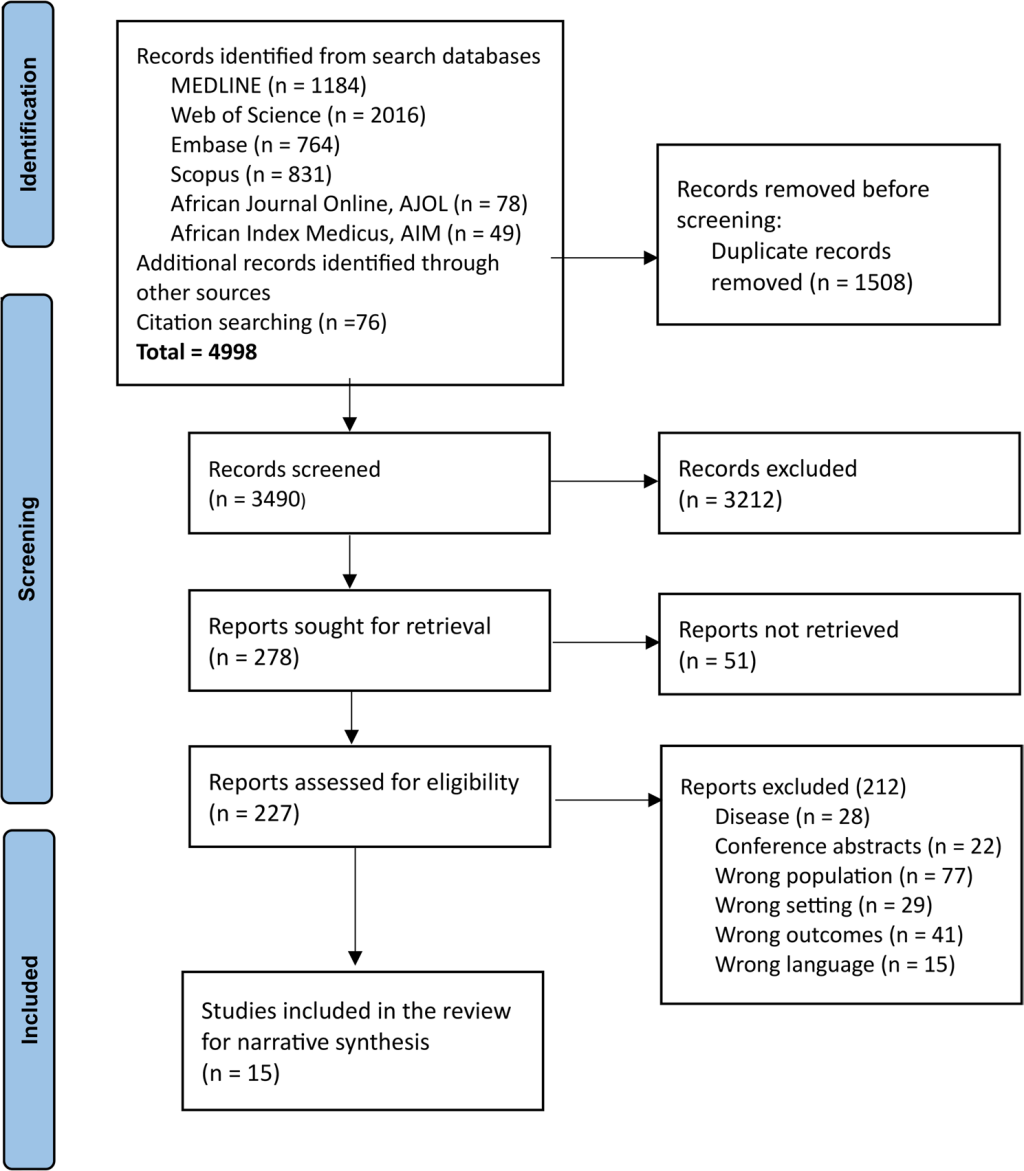
PRISMA (Preferred Reporting Items for Systematic Reviews and Meta-Analyses) flow diagram showing the selection of studies.

Study selection was reported according to the Preferred Reporting Items for Systematic Reviews and Meta-Analysis (PRISMA) [39] (see Fig. 2). The search results were transferred to the online Covidence software (available at www.covidence.org), where 2 reviewers (O.D.I., J.O.O., and E.O.W.) independently screened the titles and abstracts of all search outputs after duplicates were removed, and conflicts were resolved between O.D.I., J.O.O., and E.O.W. A fourth reviewer (V.R.) evaluated 10% of the discarded papers at both stages. Full texts of potential qualifying abstracts were retrieved and screened for relevance; only those that met the inclusion criteria were included in the review. Conflicts in full-text selection were resolved between O.D.I. and V.R., and when they persisted, were discussed with E.A.W. and resolved. 

### Data extraction

Data extraction was conducted using a prespecified Microsoft Excel spreadsheet containing the following items: authors, year of publication, country of study, sample size, population studied, sampling strategy, study design, method for evaluating nutrition status, and method for evaluating physical function. The tool was piloted with two articles prior to final extraction. The data extraction was performed independently by one researcher (O.D.I), with a 100% check by a second reviewer (V.R). To account for methodological diversity, quality, and sample variability, the study adopted a narrative synthesis [40]. A textual approach was used to summarise the included studies, focusing on methodological quality in a data extraction sheet and on relationships within and between the studies reviewed.

### Quality assessment of articles and risk of bias

All studies identified were cross-sectional observational studies. The quality of the selected studies was assessed using the Critical Appraisal Checklist for cross-sectional studies [41] from the Joanna Briggs Institute [42]. The tool considers eight methodological aspects, including: inclusion criteria, the population studied, exposure and conditions measured, identification and strategies for dealing with confounding factors, validity and reliability of outcome measurements, and statistical analysis. The tool is preferred and suitable for cross-sectional studies that report on data with prevalence [41]. The risk of bias for each article was categorised as “low risk”, “moderate risk” or “high risk” based on the tool’s point score. The quality assessment was carried out independently by the first author (O.D.I.), and 80% were checked by the second author (V.R.). In the event of any differences, they were resolved through discussion among them and E.A.W. Meta-analysis was not carried out due to heterogeneity in the study tools and variations in settings.

## Results

A total of 4998 records, including those subjected to citation checking, were identified through the search strategies, of which 1508 were duplicates (Fig. 2). Of the 3490 remaining records, 3212 were excluded following title and abstract screening, leaving 278 reports for retrieval. Of the 278 reports that were sought for retrieval, 227 reports were retrieved, while 51 reports were not retrieved. For the full-text screening, the inclusion and exclusion criteria were applied to each of the 227 articles retrieved. Only 15 studies met the criteria and were included in this review. Out of the excluded 212 articles, 28 studies were disease-related, 22 were conference abstracts, 77 studies were wrong populations, 29 studies were wrong setting (outside sub-Saharan Africa), 41 articles had wrong outcomes (nutrition status and physical function were not measured), and 15 others were wrong language.

### Characteristics of included studies

#### Study population

Of the 15 studies that met the inclusion criteria and were included in the review focused on the population of 60 years and above [25, 43–56]. The total number of participants included in the studies ranged from 100 [43] to 9736 [45]. All of the studies included participants of both sexes. See Table 2 for details.

**Table 2:** Characteristics of the studies included in the review

Reference	Year	Country	Title	Study design	Sample Size	Population Studied	Study Setting	Sampling Method	NS measures	PF measures
Adepoju et al. [25]	2021	Nigeria	Nutritional Status and Functional Capacity of the Elderly in Selected Communities in Ibadan, Oyo State	Cross-sectional	450	Men & Women 60yrs & above	Urban area	Multistage Sampling technique	BMI	BADL & IADL
Andre et al. [54]	2013	DRC	Mini Nutritional Assessment and functional capacity in community-dwelling elderly in Rural Luozi, Democratic Republic of Congo	Cross-sectional	370	Men & Women 65–88 years	Rural area	Convenient	BMI & MNA-LF	ADL & IADL
Corso et al. [45]	2025	Ghana & South Africa	Nutritional status is associated with cognition and grip strength among older adults: A 10-year longitudinal study in Ghana and South Africa	Longitudinal	9736	Men & Women 60 yrs	Urban	Multistage cluster sampling	MNA-SF	HGS
Charlton et al. [52]	2005	South Africa	Micronutrient dilution associated with added sugar intake in elderly black South African women.	Cross-sectional	285	Men & Women 60yrs & above	Urban area	Convenient	BMI	HGS, Sit-to-stand, & Timed-up-and-go
Charlton et al. [53]	2007	South Africa	The MNA, but not the DETERMINE, screening tool, is a valid indicator of nutritional status in elderly Africans	Cross-sectional	283	Men & Women 60yrs & above	Urban area	Purposive	MNA-LF, BMI	ADL, IADL, HGS, GS, Mobility balance
Chilima & Ismail [51]	2001	Malawi	Nutrition and handgrip strength of older adults in Rural Malawi	Cross-sectional	284	Men & Women 60yrs & above	Rural area	Multistage cluster sampling	BMI, MUAC, Armspan, SF	HGS, Sit-to-stand, & Timed-up-and-go
Kikafunda & Lukwago [43]	2005	Uganda	Nutritional status and functional ability of the elderly aged 60 to 90 years in the Mpigi district of central Uganda	Cross-sectional	100	Men & Women 60–90 years	Rural area	Stratified random sampling	BMI, MUAC, Armspan	ADL
Tesfaye et al. [46]	2024	Ethiopia	Nutritional status of hospitalised elderly patients in Ethiopia: a cross-sectional study of an important yet neglected problem in clinical practice	Cross-sectional	157	Men & Women 60 yrs & above	Urban	Convenient	MNA-LF	ADL
Mphwanthe et al. [47]	2025	Malawi	Risk of malnutrition, food insecurity, dietary quality, and associated factors among Malawian older adults at hospital admission: a cross-sectional study	Cross-sectional	315	Men & Women 60–93 years	Urban	Non-probability consecutive	MNA-SF, GLIM	HGS & ADL
Nyaruhucha et al. [18]	2004	Tanzania	Nutritional status, functional ability and food habits of institutionalised and non-institutionalised elderly people in Morogoro Region, Tanzania	Cross-sectional	100	Men & Women 60yrs & above	Urban area	Not reported	BMI	BADL
Olawumi et al. [50]	2021	Nigeria	Functional Correlates of Malnutrition Among Older Patients in a Primary Care Clinic in Northern Nigeria	Cross-sectional	352	Men & Women 60yrs & above	Urban area	Systematic random sampling	BMI, MNA-LF, MAC	ADL & IADL
Gabriel & Alaba [49]	2024	Nigeria	Nutritional status and functional capacity of elderly in selected communities in Yewa South, Ogun State	Cross-sectional	319	Men & Women 60 years & above	Urban	Multistage sampling	BMI	BADL & IADL
Pieterse et al. [55]	2002	Tanzania	The association between nutritional status and handgrip strength in older Rwandan refugees	Cross-sectional	444	Men & Women 60yrs & above	Urban area	Not reported	BMI, MUAC, SF	HGS
Nzeagwu & Ozougwu [48]	2019	Nigeria	Nutritional and health status of older persons in rural communities of Udi Local Government Area, Enugu State	Cross-sectional	238	Men & Women 60 yrs & above	Rural	Random sampling	BMI	ADL & IADL
Shozi et al. [56]	2022	South Africa	Relationships between physical activity, body mass index, waist circumference and handgrip strength amongst adults from the Northwest province, South Africa: The PURE study.	Cross-sectional	243	Men & women 60–70 years	Urban area	Not reported	BMI & WC	HGS

*Abbreviations*: *NS* nutritional status, *PF *physical function, *BMI *body mass index, *MNA*(LF, SF) mini nutritional assessment (LF: long form, SF: short form), *MUAC* mid-upper arm circumference, *MAC *mid-arm circumference, *WC *waist circumference, *HGS* handgrip strength, *ADL* activities of daily living, *IADL *instrumental activities of daily living, *BADL *basic activities of daily living, *GLIM *Global Leadership Initiative on Malnutrition

#### Geographical distribution

Of the 15 studies included, four studies [25, 48–50] were conducted in Nigeria, four in South Africa [45, 52, 53, 56], two each from Malawi [47, 51] and Tanzania [44, 55], and one each from Ghana [45], Democratic Republic of Congo (DRC) [54], Ethiopia [46] and Uganda [43] (See Table 2).

#### Nutritional status assessment measures

With regard to the tool that was used to assess nutritional status, four studies [25, 44, 48, 49] used BMI only, while two studies used both BMI and MNA [53, 54]. Four studies [45–47, 50] used Mini Nutrition Assessment, either short or full form (MNA), while one study [56] utilised BMI and waist circumference. Additionally, one further used the GLIM criteria in addition to the MNA-SF [47]. See Table 2 for details.

#### Physical function assessment measures

For the assessment of physical function, three studies [45, 55, 56] used handgrip strength only (HGS), while two studies used handgrip strength (HGS), sit-to-stand, and the Timed Up and Go test [51, 52]. One study used a combination of HGS, ADL and IADL to quantify participants’ physical function [47]. Three studies [43, 44, 46] used ADL only, while five studies [25, 48–50, 54] used ADL and IADL. Table 2 shows the characteristics of the studies.

### Nutrition status findings

#### Anthropometry

Of the 15 studies included, 11 [25, 43, 44, 48, 49, 51–56] reported nutritional status using the World Health Organisation’s body mass index (BMI) cutoffs. The BMI results showed that undernutrition (underweight) ranged from 1.7% [48] to 34% [54], while overweight ranged from 8.2% [25] to 38.8% [49]. Obesity was reported to range from 8% [25] to 65% [53]. Those who were reported to have a normal status ranged from 37.8% [48] to 66.7% [25]. Among the studies that reported on nutrition status using BMI, five [43, 44] reported results separately for men and women. The findings showed varying results between men and women, with the men having higher rates of undernutrition and the women having higher rates of obesity (Table 3). Some of the studies [43, 51, 56] measured body composition using other methods such as mid-upper arm circumference (MUAC) and waist circumference (WC). Details on nutritional status and physical function for all the identified papers are presented in (Table 3).

**Table 3:** Results of selected studies of nutrition status and physical function

Reference	Year	Nutrition Status Outcomes	Physical Function outcomes
Adepoju et al. [25]	2021	BMI: Underweight 17%, Overweight 8.2%, Obesity 8%, Normal weight 66.7%.	BADL: 91% functional independent, 7% moderate, 2% severe; IADL: 71% functional independent, 19% moderate, 10% severe
Andre et al. [54]	2013	BMI: Underweight 34.3%; MNA-LF: Normal 13.8%, Risk of malnutrition 57.8%, malnutrition 28.4%.	ADL: 76.5% dependent, 23.5% independent; IADL: 72.7% dependent, 27.3% independent.
Corso et al. [45]	2025	MNA-SF (Result for Ghana): Malnutrition = 9.9%, Risk of malnutrition = 48.9%, Normal = 41.2%. MNA-SF (Result for South Africa): Malnutrition = 3.6%, Risk of malnutrition = 31.1%, Normal 65.3%	Mean HGS (Ghana): 23.4 ± 10.7 kg Mean HGS (South Africa): 36.4 ± 19.6 kg
Charlton et al. [52]	2005	BMI- Women: Underweight = 2.2%, Overweight = 20%, Obese = 65%; Men: Underweight = 19.2%, Overweight = 25.5%, Obese = 14%	ADL: 8.8% dependent, 91.2% independent. No data reported on HGS
Charlton et al. [53]	2007	BMI: 65% obese, 20% overweight, MNA-LF: Malnutrition = 5.6%, Risk of malnutrition = 50.4%, Normal = 44%	Mean HGS: Men = 24.1 ± 7.7 kg; women = 15.1 ± 5.3 kg.
Chilima & Ismail [51]	2001	BMI: Undernutrition = 17.95%	Mean HGS: men = 28.0 ± 5.9 kg; women = 21.7 ± 4.5 kg
Kikafunda & Lukwago [43]	2005	BMI: Underweight = 33.4% (total), Men = 32%, Women = 68%; Normal = 58.0%, Overweight = 8.6%.	ADL: 67% dependent, 33% independent
Tesfaye et al. [46]	2024	MNA-LF: Malnutrition = 81%, Risk of malnutrition = 17%, Normal = 2%.	ADL: 72% dependent, 28% independent
Mphwanthe et al. [47]	2025	MNA-SF: Malnutrition = 40.3%, Risk of malnutrition = 39.7%, Normal = 20%. GLIM-criteria: Malnutrition = 75%	ADL: 37.1% dependent, 62.9 independent. HGS (kg): Men(< 27 kg) = 97.8%, women(< 16 kg) = 74.8%
Nyaruhucha et al. [18]	2004	BMI: Underweight = 30.1% (total) women = 22.7%, Men = 39.3%.	ADL: 21% dependent, 79% independent
Olawumi et al. [50]	2021	MNA-LF: Malnutrition = 25.9%, Risk of malnutrition = 53.1%, normal = 21%.	ADL: 21.6% dependent, 78.4% independent. IADL: 32.4% dependent, 67.6% independent.
Gabriel & Alaba [49]	2024	BMI: Underweight = 3.1%, Overweight = 38.8%, Normal = 45.9%, Obese = 12.2%.	BADL and IADL: 12.2% dependent, 87.8% independent
Pieterse et al. [55]	2002	BMI: Undernutrition 23.2% for men, 15.0% for women	Mean HGS: men = 30 ± 6.7 kg; women = 22 ± 5.1 kg
Nzeagwu & Ozougwu [48]	2019	BMI: Underweight = 1.7%, Normal = 37.8%, Overweight = 34.5%, Obese = 26.1%	ADL: 7.6% dependent, 92.4% independent. IADL: 27.7% dependent, 72.3% independent
Shozi et al. [56]	2022	Mean BMI: men = 22.16 ± 5.14 kg/m^2^, women = 27.46 ± 6.06 kg/m^2^; Underweight = 9.0%. Mean WC: men = 83.20 ± 12.96 cm, women = 90.89 ± 12.97 cm	Mean HGS: men = 30.28 ± 8.69 kg; women = 23.27 ± 6.45 kg

*Abbreviations*: *NS *nutritional status, *PF *physical function, *BMI *body mass index, *MNA *mini nutritional assessment, *MUAC *mid-upper arm circumference, *MAC *mid-arm circumference, *WC *waist circumference, *HGS *handgrip strength, *ADL *activities of daily living, *IADL* instrumental activities of daily living, *BADL *basic activities of daily living, kg-kilogram, cm centimetre, %- percentage

#### Mini-nutritional assessment and global leadership initiative on malnutrition (GLIM) criteria

Six studies [45–47, 50, 53, 54] used the MNA with one of them, adding the GLIM criteria [47] for reporting their findings. Two of the six studies used the short form [45, 47], while the others used the full version [46, 50, 53, 54]. Malnutrition with MNA-SF was reported to range from 3.6% [45] to 40.3% [47] while the MNA-LF was between 5.6% [53] to 81% [46]. Those at risk of malnutrition were found to be between 31.1% [45] to 57.8% and those with normal nutritional status ranged from 2% [46] to 65.3% [45]. According to the study that used GLIM criteria, malnutrition was reported at 75% [47]. See Table 3 for details.

#### Physical function findings

Of the 15 included studies that reported findings, ten studies [25, 43, 44, 46–50, 52, 54] used ADL and IADL. The ADL showed dependency levels for different activities or functions ranging from 7.6% to 76.5%, and the IADL ranged from 27.7% to 72.7% (see Table 3). Of the other studies that assessed physical function, six studies [45, 47, 51, 53, 55, 56] reported the findings with HGS. The HGS was higher in men than in women in most cases (see Table 3).

#### Quality assessment and risk of bias of included studies

**Fig. 3:**
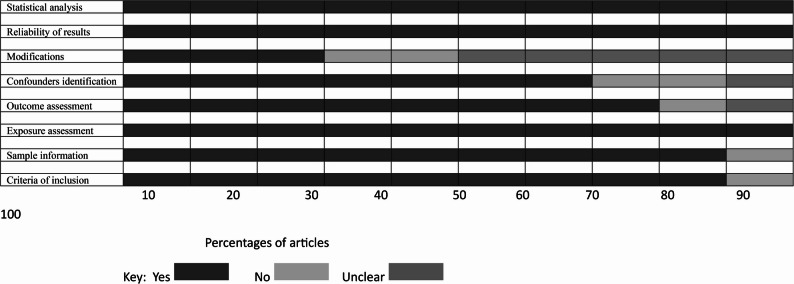
Quality assessment and risk of bias of studies using critical appraisal checklist for observational and analytical studies from the joanna briggs institute (JBI)

The methodological quality and risk of bias (RoB) for the included studies are summarised in (Fig. 3). Overall, the evidence base demonstrated high methodological integrity, with the majority of studies exhibiting low to moderate risk of bias. Quantitative assessment revealed that 60% (*n* = 9) of the included studies were classified as having a low risk of bias, while 26.6% (*n* = 4) were classified as having a moderate risk. A minority of the literature (13.4%, *n* = 2) was identified as high risk, primarily due to limitations in specific methodological domains. The internal validity of the evidence was strongest in exposure assessment, statistical rigour, and the reliability of reported results, all of which were consistently well documented across the cohort. In contrast, the most frequent methodological weaknesses were identified as: confounder adjustment and inadequate control for extraneous variables. Specifically, three studies [45–47] achieved a perfect score by meeting all pre-specified criteria, while nine studies [18, 25, 43, 50–52, 54–56] demonstrated high adherence, meeting 87.5% of the assessment items. Although a systematic assessment of quality was conducted, a formal evaluation of the certainty of evidence—such as the GRADE (Grading of Recommendations Assessment, Development and Evaluation) framework—was not pursued. This decision was predicated on substantial heterogeneity across study designs, diverse populations, and varied metrics used to define outcomes, which precluded a meaningful pooled certainty analysis.

## Discussion

This systematic review synthesised evidence from 15 studies across eight sub-Saharan African (SSA) countries, revealing a significant but complex association between undernutrition and diminished physical function in older adults. We observed a high prevalence of undernutrition (up to 34%) and risk of malnutrition (up to 57.8%), figures that notably exceed reported rates for mid-adults in the same region (2.7%–19.5%) [57, 58]. Crucially, our findings indicate that poor nutritional status is positively correlated with declines in handgrip strength (HGS) and impairments in both activities of daily living (ADL) and instrumental activities of daily living (IADL), although this directionality cannot be assumed due to the cross-sectional design of the included studies (see Table 4).

**Table 4:** Summary of findings on the relationship between nutrition status and physical function

Reference	Summary of findings for the link between NS & PF	Measure of association
Adepoju et al. [25]	Nutrition status was significantly associated with physical function in aspects of ADL and IADL.	Χ^2^ = 45.91 (*p* < 0.00)
Andre et al. [54]	Malnutrition was high, with reduced functional ability	Did not report figures
*Corso et al. [45]	A positive association was found between nutritional status and physical function	β = 2.43 (*p* < 0.0001)
Charlton et al. [52]	Physical function declined with poor nutritional status.	*r* = 0.165 (*p* < 0.05)
Charlton et al. [53]	The study showed a positive correlation between physical function and nutritional status	*r* = 0.156 (*p* < 0.05)
Chilima & Ismail [51]	The study revealed that poor handgrip strength and physical function were correlated with poor nutritional status	β = 0.408 (*p* < 0.000)
Kikafunda & Lukwago [43]	The study found a high level of undernutrition correlated with poor functional ability in the studied population.	Coefficients not reported; *p* < 0.05
Tesfaye et al. [46]	No relationship was observed between nutritional status and physical function	AOR 3.689; 95% CI: 1.190, 11.433 (*p* < 0.024)
Mphwanthe et al. [47]	Malnutrition was significantly observed to be associated with reduced functional independence	AOR 5.182; 95% CI: 1.469–18.279 (*p* < 0.011)
Nyaruhucha et al. [18]	The difference was observed in the nutritional status between the institutionalised and non-institutionalised participants, as well as in the inability to perform daily activities of living, such as feeding and mobility.	Did not report figures
Olawumi et al. [50]	The study showed that functional dependence was a determinant of malnutrition. The association between nutrition status and the physical function of the participants was significant	OR = 14.706, 95% CI = 1.263–3.349, (*p* = 0.032)
Gabriel & Alaba [49]	No correlation was observed between physical function and nutritional status	Did not report figures
Pieterse et al. [55]	The study revealed that poor handgrip strength was associated with poor nutritional status	β = 0.262 (*p* < 0.001)
Nzeagwu & Ozougwu [48]	No relationship was observed between nutritional status and physical function among older adults studied	*r* = -0.085 (*p* > 0.191)
Shozi et al. [56]	The study revealed that nutritional status was correlated with poor upper limb muscle strength	*r* = 0.24 (*p* < 0.001)

Causality inference cannot be assumed, as 97% of the included studies were cross-sectional observational studies

*Corso et al. was a longitudinal study

*Abbreviations*: *NS *nutritional status, *PF *physical function, *ADL *activities of daily living, *IADL *instrumental activities of daily living

The wide range in undernutrition prevalence (1.7% in urban South Africa to 30% in Tanzania) highlights a profound socio-economic gradient. The relative food security and healthcare infrastructure in South Africa likely provide a protective effect compared to lower-income nations like Tanzania or Nigeria [35]. Furthermore, a distinct rural-urban divide was evident. Studies in rural Ghana, Uganda, and the DRC reported higher undernutrition rates, likely driven by limited access to food, lower nutritional literacy, and the absence of government social safety nets [43, 54]. These results align with global trends where low-income status dictates dietary quality, thereby exacerbating food insecurity and reducing the nutritional well-being of ageing populations [59–62].

While obesity is traditionally more prevalent among African women [63], our findings show that undernutrition and malnutrition risk remain disproportionately high in this group. Interestingly, despite lower nutritional status, the mean HGS of 23.27 kg in African women in Malawi, Tanzania, and South Africa was comparable to that of 19.4 kg in women from high-income countries [64–66]. This “resilience” in muscle strength may reflect the high levels of lifelong physical labour and domestic chores undertaken by older women in SSA. In contrast, men’s HGS aligned with low- and medium-income countries (LMICs) averages but remained lower than Western benchmarks, potentially signalling a premature onset of ageing due to chronic illness and strenuous labour [51, 53, 56].

The correlation between undernutrition and functional decline appears bidirectional. Impairments in physical function—specifically in food preparation (33.1%), shopping (32.4%), and mobility (33.3%) — directly impede older adults’ ability to maintain adequate nutrition [18, 25, 53]. Conversely, inadequate nutrition results in increased morbidity and decreased mobility [67]. While HGS emerged as a reliable proxy for physical function, its correlation with body mass index (BMI) was inconsistent across the literature. While some studies suggest HGS increases with BMI [56, 68], others found no definitive link [69], likely due to the “obesity paradox” or the presence of sarcopenic obesity, where high BMI masks underlying muscle wasting [56, 70].

A significant finding of this review is the limitation of current assessment tools. BMI remains the most utilised measure of nutritional status [71], yet its validity in older adult populations is contested. The use of WHO cutoffs (e.g., BMI < 18.5 kg/m^2^) may lead to an underestimation of undernutrition [71]; recent evidence suggests a higher threshold of ≤ 22 kg/m^2^ is more appropriate for older adults to account for height loss and body composition shifts [72, 73]. The MNA tool proved more comprehensive in clinical settings, though its reliance on questionnaires introduces inherent subjectivity and recall bias [53, 74]. Moving forward, adopting the GLIM criteria, which combine phenotypic and etiologic measures, may provide the necessary consensus for diagnosing malnutrition in this demographic [21]. Undernutrition is a significant driver of functional decline among older adults in Sub-Saharan Africa (Table 4), particularly in rural and low-income settings. There is an urgent need for longitudinal research to establish the causal pathway between nutritional interventions and physical preservation. Policymakers should prioritise multifaceted assessment strategies rather than BMI alone to identify and support at-risk older adults in this rapidly ageing region.v

This is the first systematic review to specifically examine the intersection of nutrition and physical function among older adults in SSA. A major strength is the inclusion of objective measures of physical performance, which reduces the risk of reporting bias. However, several limitations must be noted: Study Design: 14 of the 15 studies were cross-sectional, precluding any definitive inferences regarding causality. Heterogeneity: Variations in sample sizes (*n* = 100–9736), sampling methods, recruitment settings, and assessment tools made a meta-analysis unfeasible. Search Strategy: Restricting the search to English-language articles, conducting the initial search by a single researcher, and excluding specific sub-national state terms may have led to the omission of relevant data. A certainty assessment of the evidence for primary outcomes was also not carried out in this study due to the high heterogeneity of the studies and the narrative nature of the synthesis.

## Conclusion

This systematic review underscores that the triple burden of malnutrition—encompassing undernutrition, overnutrition, and malnutrition risk—persists as a critical public health challenge among older adults in Sub-Saharan Africa. The evidence consistently identifies a significant correlation between nutritional status and functional outcomes, specifically muscle strength and independence in both activities of daily living (ADLs) and instrumental activities of daily living (IADLs). However, the current literature is predominantly cross-sectional. To move beyond association and establish definitive causality, future research must prioritise longitudinal designs. These findings call for the integration of routine nutritional screening into geriatric care frameworks across the region to preserve functional autonomy in an ageing population.

### Key takeaways

- Older adults in Sub-Saharan Africa face a “triple burden” of malnutrition—undernutrition, overnutrition, and significant malnutrition risk—highlighting a major, unaddressed public health gap.
- Nutritional status is a critical determinant of physical autonomy; poor nutrition is consistently correlated with diminished muscle strength and a reduced ability to perform activities of daily living (ADLs/IADLs).
- While the association between nutrition and physical function is clear, current evidence is limited by cross-sectional data. Longitudinal research is essential to determine if malnutrition is a precursor to or a result of functional decline.
- There is an urgent need to integrate standardised nutritional screening and interventions into primary healthcare frameworks across Sub-Saharan Africa to support healthy ageing.

## Supplementary Information

Supplementary Material 1.

## Data Availability

This systematic review relies on data obtained from studies published in publicly accessible literature. All data generated or analysed during this investigation are encompassed within this published article, including its tables, figures, and supplementary file. Further details can be obtained from the corresponding author upon a justifiable request.

## References

[1] World Health O. Decade of healthy ageing: baseline report. World Health Organization; 2021.

[2] Allen B, Saunders J. Malnutrition and undernutrition: causes, consequences, assessment and management. Medicine. 2023;51(7):461–8.

[3] Salari N, Darvishi N, Bartina Y, Keshavarzi F, Hosseinian-Far M, Mohammadi M. Global prevalence of malnutrition in older adults: a comprehensive systematic review and meta-analysis. Public Health Pract. 2025;9:100583. 10.1016/j.puhip.2025.100583 PMC11780955 39885903

[4] Ngo J, Ortiz-Andrellucchi A, Serra-Majem L, Malnutrition. Concept, Classification and Magnitude. In: Caballero B, Finglas PM, Toldrá F, editors. Encyclopedia of food and health. Oxford: Academic; 2016. pp. 610–30.

[5] Deer RR, Hosein E, Mera A, Howe K, Goodlett S, Robertson N, et al. Dietary Intake patterns of community-dwelling older adults after acute hospitalization. J Gerontol Biol Sci Med Sci. 2022;77(1):140–7. 10.1093/gerona/glab232 PMC8923293 34410002

[6] Hernández-Galiot A, Goñi I. Quality of life and risk of malnutrition in a home-dwelling population over 75 years old. Nutrition. 2017;35:81–6. 28241994 10.1016/j.nut.2016.10.013

[7] Ferdous T, Kabir ZN, Wahlin A, Streatfield K, Cederholm T. The multidimensional background of malnutrition among rural older individuals in Bangladesh–a challenge for the Millennium Development Goal. Public Health Nutr. 2009;12(12):2270–8. 19257922 10.1017/S1368980009005096

[8] Valentini A, Federici M, Cianfarani MA, Tarantino U, Bertoli A. Frailty and nutritional status in older people: the Mini Nutritional Assessment as a screening tool for the identification of frail subjects. Clin Interv Aging. 2018;13:1237–44. 30034227 10.2147/CIA.S164174 PMC6047619

[9] Krondl M, Coleman P, Lau D. Helping older adults meet nutritional challenges. J Nutr Elder. 2008;27(3–4):205–20. 19042572 10.1080/01639360802261755

[10] Ahmed T, Haboubi N. Assessment and management of nutrition in older people and its importance to health. Clin Interv Aging. 2010;5:207–16. 20711440 10.2147/cia.s9664 PMC2920201

[11] Landi F, Calvani R, Tosato M, Martone AM, Ortolani E, Savera G, et al. Anorexia of aging: risk factors, consequences, and potential treatments. Nutrients. 2016;8(2):69. 26828516 10.3390/nu8020069 PMC4772033

[12] Barrientos A, Gorman M, Heslop A. Old age poverty in developing countries: contributions and dependence in later life. World Dev. 2003;31(3):555–70.

[13] Feng W. Social exclusion of the elderly in contemporary China: One empirical study based on the surveys in six provinces. China Development Research Foundation; 2011.

[14] Ahmad MH, Salleh R, Siew Man C, Pardi M, Che Abdul Rahim N, Shahril N, et al. Malnutrition among the Elderly in Malaysia and its associated factors: findings from the national health and morbidity survey 2018. J Nutr Metab. 2021;2021:6639935. 33953978 10.1155/2021/6639935 PMC8057910

[15] Yang Z, Hall AG. The financial burden of overweight and obesity among elderly Americans: the dynamics of weight, longevity, and health care cost. Health Serv Res. 2008;43(3):849–68. 18454771 10.1111/j.1475-6773.2007.00801.x PMC2442233

[16] Beaudart C, Rolland Y, Cruz-Jentoft AJ, Bauer JM, Sieber C, Cooper C, et al. Assessment of muscle function and physical performance in daily clinical practice: a position paper endorsed by the european society for clinical and economic aspects of osteoporosis, osteoarthritis and musculoskeletal diseases (ESCEO). Calcif Tissue Int. 2019;105(1):1–14. 30972475 10.1007/s00223-019-00545-w

[17] Norman K, Haß U, Pirlich M. Malnutrition in older adults-recent advances and remaining challenges. Nutrients. 2021;13(8). 10.3390/nu13082764 PMC8399049 34444924

[18] Nyaruhucha CN, Msuya JM, Matrida E. Nutritional status, functional ability and food habits of institutionalised and non-institutionalised elderly people in Morogoro Region, Tanzania. East Afr Med J. 2004;81(5):248–53. 15508339 10.4314/eamj.v81i5.9168

[19] Huntley J, Ostfeld AM, Taylor JO, Wallace RB, Blazer D, Berkman LF, et al. Established populations for epidemiologic studies of the elderly: study design and methodology. Aging Clin Exp Res. 1993;5(1):27–37. 10.1007/BF03324123 8481423

[20] Podsiadlo D, Richardson S. The timed Up & Go: a test of basic functional mobility for frail elderly persons. J Am Geriatr Soc. 1991;39(2):142–8. 1991946 10.1111/j.1532-5415.1991.tb01616.x

[21] Cederholm T, Jensen GL, Correia MITD, Gonzalez MC, Fukushima R, Pisprasert V, et al. The GLIM consensus approach to diagnosis of malnutrition: A 5-year update. Clin Nutr. 2025;49:11–20. 40222089 10.1016/j.clnu.2025.03.018

[22] Cruz-Jentoft AJ, Kiesswetter E, Drey M, Sieber CC. Nutrition, frailty, and sarcopenia. Aging Clin Exp Res. 2017;29(1):43–8. 28155181 10.1007/s40520-016-0709-0

[23] Asp M, Simonsson B, Larm P, Molarius A. Physical mobility, physical activity, and obesity among elderly: findings from a large population-based Swedish survey. Public Health. 2017;147:84–91. 28404503 10.1016/j.puhe.2017.01.032

[24] White JV, Guenter P, Jensen G, Malone A, Schofield M, Force AMT, et al. Consensus statement of the Academy of Nutrition and Dietetics/American Society for Parenteral and Enteral Nutrition: characteristics recommended for the identification and documentation of adult malnutrition (undernutrition). J Acad Nutr Dietetics. 2012;112(5):730–8. 10.1016/j.jand.2012.03.012 22709779

[25] Adepoju A, Olayiwola I, Onabanjo O, Lasode O. Nutritional Status and Functional Capacity of Elderly in Selected Communities in Ibadan, Oyo State. Nigerian J Nutritional Sci. 2021;42(2):41–52.

[26] Saki T, Rashidi F, Mamene M, Azadi H, Azadi A. Healthy aging from the perspective of older adults: a descriptive qualitative study. 2024.

[27] Alwarawrah Y, Kiernan K, MacIver NJ. Changes in nutritional status impact immune cell metabolism and function. Front Immunol. 2018;9:1055. 29868016 10.3389/fimmu.2018.01055 PMC5968375

[28] Rodríguez-Mañas L, Murray R, Glencorse C, Sulo S. Good nutrition across the lifespan is foundational for healthy aging and sustainable development. Front Nutr. 2023;9:1113060. 36761990 10.3389/fnut.2022.1113060 PMC9902887

[29] Struijk EA, Hagan KA, Fung TT, Hu FB, Rodríguez-Artalejo F, Lopez-Garcia E. Diet quality and risk of frailty among older women in the Nurses’ Health Study. Am J Clin Nutr. 2020;111(4):877–83. 32091575 10.1093/ajcn/nqaa028 PMC7138663

[30] Ge L, Li R, Yap CW. The interplay of nutritional status and physical function on health outcomes in older adults: a longitudinal study in Singapore. Am J Clin Nutr. 2025. 10.1016/j.ajcnut.2025.07.014 40706959

[31] Ge L, Yap CW, Heng BH. Association of nutritional status with physical function and disability in community-dwelling older adults: a longitudinal data analysis. J Nutr Gerontol Geriatr. 2020;39(2):131–42. 32048552 10.1080/21551197.2020.1725711

[32] Asamane EA, Greig CA, Thompson JL. The association between nutrient intake, nutritional status and physical function of community-dwelling ethnically diverse older adults. BMC Nutr. 2020;6(1):36. 32864152 10.1186/s40795-020-00363-6 PMC7447572

[33] Fried LP, Tangen CM, Walston J, Newman AB, Hirsch C, Gottdiener J, et al. Frailty in older adults: evidence for a phenotype. journals Gerontol Ser a: Biol Sci Med Sci. 2001;56(3):M146–57. 10.1093/gerona/56.3.m146 11253156

[34] Cederholm T, Jensen GL, Correia M, Gonzalez MC, Fukushima R, Higashiguchi T, et al. GLIM criteria for the diagnosis of malnutrition - A consensus report from the global clinical nutrition community. J Cachexia Sarcopenia Muscle. 2019;10(1):207–17. 30920778 10.1002/jcsm.12383 PMC6438340

[35] Obeng P, Kyereh HK, Sarfo JO, Ansah EW, Attafuah PYA. Nutritional status and associated factors of older persons in sub-Saharan Africa: a scoping review. BMC Geriatr. 2022;22(1). 10.1186/s12877-022-03062-y PMC9097054 35545755

[36] Seid AM, Fentahun N. Prevalence of malnutrition among old people in Africa: systematic review and meta-analysis. BMJ Open. 2022;12(11). 10.1136/bmjopen-2022-065197 PMC9716798 36450428

[37] Mezgebu GS, Petros L, Alemayew E, Abebaw G, Feleke FW. Magnitude of undernutrition and its association with dietary diversity among older persons in Ethiopia: a systematic review and meta-analysis, 2023. J Nutritional Sci. 2023;12. 10.1017/jns.2023.84 PMC10523292 37771505

[38] Yisak H, Zemene MA, Arage G, Demelash AT, Anley DT, Ewunetei A et al. Undernutrition and associated factors among older adults in Ethiopia: systematic review and meta-analysis. BMJ Open. 2023;13(1). 10.1136/bmjopen-2022-062845 PMC9884872 36693689

[39] Page MJ, McKenzie JE, Bossuyt PM, Boutron I, Hoffmann TC, Mulrow CD et al. The PRISMA 2020 statement: an updated guideline for reporting systematic reviews. BMJ. 2021;372. 10.1136/bmj.n71 PMC8005924 33782057

[40] Popay J, Roberts H, Sowden A, Petticrew M, Arai L, Rodgers M, et al. Guidance on the conduct of narrative synthesis in systematic reviews. A product from the ESRC. methods programme Version. 2006;1(1):b92.

[41] Ma L-L, Wang Y-Y, Yang Z-H, Huang D, Weng H, Zeng X-T. Methodological quality (risk of bias) assessment tools for primary and secondary medical studies: what are they and which is better? Military Med Res. 2020;7:1–11. 10.1186/s40779-020-00238-8 PMC7049186 32111253

[42] Pereira MHQ, Pereira MLAS, Campos GC, Molina MCB. Food insecurity and nutritional status among older adults: a systematic review. Nutr Rev. 2022;80(4):631–44. 34338784 10.1093/nutrit/nuab044

[43] Kikafunda JK, Lukwago FB. Nutritional status and functional ability of the elderly aged 60 to 90 years in the Mpigi district of central Uganda. Nutrition. 2005;21(1):59–66. 15661479 10.1016/j.nut.2004.09.009

[44] Nyaruhucha CNM, Msuya JM, Matrida E. Nutritional status, functional ability and food habits of institutionalised and non-institutionalised elderly people in Morogoro Region, Tanzania. East Afr Med J. 2004;81(5):248–53. 15508339 10.4314/eamj.v81i5.9168

[45] Corso B, Awuviry-Newton K, Appiah SCY, Doh D, Kowal P, Charlton KE. Nutritional status is associated with cognition and grip strength among older adults: A 10-y longitudinal study in Ghana and South Africa., Nutrition. (Burbank, Los Angeles County, Calif). 2025;136:112798. 10.1016/j.nut.2025.112798 40381254

[46] Tesfaye BT, Yizengaw MA, Birhanu TE, Bosho DD. Nutritional status of hospitalized elderly patients in Ethiopia: a cross-sectional study of an important yet neglected problem in clinical practice. Front Nutr. 2024;10:1227840. 38260070 10.3389/fnut.2023.1227840 PMC10800825

[47] Mphwanthe G, Reynolds C, Corish C, Mndoliro L, Columbus T, Misolo J, et al. Risk of malnutrition, food insecurity, dietary quality, and associated factors among Malawian older adults at hospital admission: a cross-sectional study. BMC Geriatr. 2025;25(1):767. 41073896 10.1186/s12877-025-06463-x PMC12512422

[48] Nzeagwu OC, Ozougwu CB. Nutritional and health status of older persons aged ≥ 60 years in rural communities of udi local government area, enugu state, Nigeria. J Dietitians Association Nigeria. 2019;10:40–51.

[49] Gabriel E, Alaba K. Nutritional status and functional capacity of elderly in selected communities in Yewa South, Ogun State. Fed Polytechnic Ilaro J Pure Appl Sci. 2024;6(1).

[50] Olawumi AL, Grema BA, Suleiman AK, Omeiza YS, Michael GC. Functional correlates of malnutrition among older patients in a primary care clinic in Northern, Nigeria: A Cross-Sectional Study. Nigerian J Basic Clin Sci. 2021;18(2):127–33.

[51] Chilima DM, Ismail SJ. Nutrition and handgrip strength of older adults in rural Malawi. Public Health Nutr. 2001;4(1):11–7. 11255491 10.1079/phn200050

[52] Charlton KE, Kolbe-Alexander TL, Nel JH. Micronutrient dilution associated with added sugar intake in elderly black South African women. Eur J Clin Nutr. 2005;59(9):1030–42. 16015273 10.1038/sj.ejcn.1602208

[53] Charlton KE, Kolbe-Alexander TL, Nel JH. The MNA, but not the DETERMINE, screening tool is a valid indicator of nutritional status in elderly Africans. Nutrition. 2007;23(7–8):533–42. 17570641 10.1016/j.nut.2007.04.015

[54] Andre MB, Dumavibhat N, Ngatu NR, Eitoku M, Hirota R, Suganuma N. Mini Nutritional Assessment and functional capacity in community-dwelling elderly in rural Luozi. Democratic Repub Congo Geriatr Gerontol Int. 2013;13(1):35–42. 10.1111/j.1447-0594.2012.00852.x 22530787

[55] Pieterse S, Manandhar M, Ismail S. The association between nutritional status and handgrip strength in older Rwandan refugees. Eur J Clin Nutr. 2002;56(10):933–9. 12373611 10.1038/sj.ejcn.1601443

[56] Shozi S, Monyeki MA, Moss SJ, Pienaar C. Relationships between physical activity, body mass index, waist circumference and handgrip strength amongst adults from the North West province, South Africa: The PURE study. Afr J Prim health care family Med. 2022;14(1):e1–11. 10.4102/phcfm.v14i1.3206 PMC9210178 35695439

[57] Ikoona EN, Toure MA, Njenga A, Namulemo L, Kaluya R, Kamara K, et al. Prevalence and factors associated with underweight among 15–49-year-old women in Sierra Leone: a secondary data analysis of Sierra Leone demographic health survey of 2019. BMC Womens Health. 2023;23(1):192. 37085835 10.1186/s12905-023-02358-4 PMC10122406

[58] Letamo G, Navaneetham K. Prevalence and determinants of adult under-nutrition in Botswana. PLoS ONE. 2014;9(7):e102675. 25054546 10.1371/journal.pone.0102675 PMC4108334

[59] Cereda E, Pedrolli C, Klersy C, Bonardi C, Quarleri L, Cappello S, et al. Nutritional status in older persons according to healthcare setting: a systematic review and meta-analysis of prevalence data using MNA^®^. Clin Nutr. 2016;35(6):1282–90. 27086194 10.1016/j.clnu.2016.03.008

[60] Andretti B, Vieites Y, Elmor L, Andrade EB. How Socioeconomic Status Shapes Food Preferences and Perceptions. J Mark. 2025;89(6):33–56.

[61] Nazri NS, Vanoh D, Leng SK. Malnutrition, low diet quality and its risk factors among older adults with low socio-economic status: a scoping review. Nutr Res Rev. 2021;34(1):107–16. 32727634 10.1017/S0954422420000189

[62] Kohanmoo A, Hashemzadeh M, Teymouri M, Zare M, Akhlaghi M. Food insecurity is associated with low diet quality and unhealthy cooking and eating habits in Iranian women. J Health Popul Nutr. 2024;43(1):42. 38486251 10.1186/s41043-024-00533-3 PMC10941397

[63] Cheserek MJ, Tuitoek PJ, Waudo JN, Msuya JM, Kikafunda JK. Anthropometric characteristics and nutritional status of older adults in the Lake Victoria Basin of East Africa: Region, sex, and age differences. South Afr J Clin Nutr. 2012;25(2):67–72.

[64] Wearing J, Konings P, Stokes M, de Bruin ED. Handgrip strength in old and oldest old Swiss adults–a cross-sectional study. BMC Geriatr. 2018;18(1):266. 30400825 10.1186/s12877-018-0959-0 PMC6219188

[65] Qaisar R, Hussain MA, Franzese F, Karim A, Ahmad F, Awad A, et al. Predictors of the onset of low handgrip strength in Europe: a longitudinal study of 42,183 older adults from 15 countries. Aging Clin Exp Res. 2024;36(1):162. 39110364 10.1007/s40520-024-02800-z PMC11306649

[66] Park H, Kim D, Jeong H-S, Jang S. Grip Strength as an Indicator of Health in Elderly Females. Healthc [Internet]. 2025;13(10):1127. p.]. 10.3390/healthcare13101127 PMC12111805 40427964

[67] Krishnamoorthy Y, Vijayageetha M, Saya GK. Validation and reliability assessment of the mini-nutritional assessment–short form questionnaire among older adults in South India. Indian J Community Med. 2021;46(1):70–4. 34035580 10.4103/ijcm.IJCM_208_20 PMC8117899

[68] Hardy R, Cooper R, Aihie Sayer A, Ben-Shlomo Y, Cooper C, Deary IJ, et al. Body mass index, muscle strength and physical performance in older adults from eight cohort studies: the HALCyon programme. PLoS ONE. 2013;8(2):e56483. 23437142 10.1371/journal.pone.0056483 PMC3577921

[69] Kim CR, Jeon Y-J, Jeong T. Risk factors associated with low handgrip strength in the older Korean population. PLoS ONE. 2019;14(3):e0214612. 30921399 10.1371/journal.pone.0214612 PMC6438516

[70] Morgan PT, Smeuninx B, Breen L. Exploring the impact of obesity on skeletal muscle function in older age. Front Nutr. 2020;7:569904. 33335909 10.3389/fnut.2020.569904 PMC7736105

[71] De Lorenzo A, Itani L, El Ghoch M, Gualtieri P, Frank G, Raffaelli G et al. Difference in body composition patterns between age groups in italian individuals with overweight and obesity: when bmi becomes a misleading tool in nutritional settings. Nutrients. 2024; 16(15). 10.3390/nu16152415 PMC11314337 39125296

[72] Volkert D, Beck AM, Cederholm T, Cruz-Jentoft A, Goisser S, Hooper L, et al. ESPEN guideline on clinical nutrition and hydration in geriatrics. Clin Nutr. 2019;38(1):10–47. 30005900 10.1016/j.clnu.2018.05.024

[73] Winter JE, MacInnis RJ, Wattanapenpaiboon N, Nowson CA. BMI and all-cause mortality in older adults: a meta-analysis123. Am J Clin Nutr. 2014;99(4):875–90. 24452240 10.3945/ajcn.113.068122

[74] Ozturk Y, Sarikaya D, Emin Kuyumcu M, Yesil Y, Koca M, Guner Oytun M, et al. Comparison of mini nutritional assessment-short and long form to predict all-cause mortality up to 7 years in geriatric outpatients. Nutr Clin Pract. 2022;37(6):1418–28. 35678359 10.1002/ncp.10878

