# Supplementary material for: Nutritional status and physical function of older adults living in sub-Saharan Africa: a systematic review
Source: BMC Nutr. 2026 May 11;12:126. doi: 10.1186/s40795-026-01351-y (PMC13335267; doi:10.1186/s40795-026-01351-y)
Supplement: Supplementary file 1 — Supplementary Material 1. [file 40795_2026_1351_MOESM1_ESM.docx]

**Supplementary file**

Quality appraisal scoring for studies included using the JB Griggs critical appraisal tool for analytical cross-sectional studies.

| Reference | Criteria Inclusion | Sample information | Exposure measurement | Outcome Assessment | Confounders identification | Modification | Reliability of result | Statistical analysis | Overall risk assessment | QA Score |
| --- | --- | --- | --- | --- | --- | --- | --- | --- | --- | --- |
| Adepoju et al., 2021 | Yes | Yes | Yes | Yes | Yes | No | Yes | Yes | Low | +7/8 |
| Andre et al., 2013 | Yes | Yes | Yes | Yes | Yes | Unclear | Yes | Yes | Low | +7/8 |
| Corso et al., 2025 | Yes | Yes | Yes | Yes | Yes | Yes | Yes | Yes | Low | +8/8 |
| Charlton et al., 2005 | Yes | Yes | Yes | Yes | Yes | Unclear | Yes | Yes | Low | +7/8 |
| Charlton et al., 2007 | Yes | Yes | Yes | Yes | Unclear | Unclear | Yes | Yes | Moderate | +6/8 |
| Chilima and Ismail 2001 | Yes | Yes | Yes | Yes | Yes | Unclear | Yes | Yes | Low | +7/8 |
| Kikafunda and Lukwago 2005 | Yes | Yes | Yes | Yes | Yes | Unclear | Yes | Yes | Low | +7/8 |
| Tesfaye et al., 2024 | Yes | Yes | Yes | Yes | Yes | Yes | Yes | Yes | Low | +8/8 |
| Mphwanthe et al., 2025 | Yes | Yes | Yes | Yes | Yes | Yes | Yes | Yes | Low | +8/8 |
| Nyaruhucha et al., 2004 | No | Yes | Yes | Yes | Yes | Unclear | Yes | Yes | Moderate | +7/8 |
| Olawumi et al., 2021 | Yes | Yes | Yes | Yes | Yes | Unclear | Yes | Yes | Low | +7/8 |
| Gabriel & Alaba, 2024 | Yes | Yes | Yes | Yes | No | No | Y | Yes | High | +5/8 |
| Pieterse et al., 2002 | Yes | No | Yes | Yes | Yes | Unclear | Yes | Yes | Moderate | +7/8 |
| Nzeagwu & Ozougwu, 2019 | Yes | Yes | Yes | Yes | No | No | Y | Yes | High | +5/8 |
| Shozi et al., 2022 | Yes | Yes | Yes | Yes | Yes | Unclear | Yes | Yes | Moderate | +7/8 |

**Search Strategy from Medline via OvidSP**

1. adult/ or exp aged/ or exp middle-aged/ #7798424
2. exp "Aged, 80 and over"/ or exp Ageing/ or exp Adult/ #21341730
3. ("older adults" or "older persons" or geriatric or elderly or ageing).mp. [mp=title, book title, abstract, original title, name of substance word, subject heading word, floating sub-heading word, keyword heading word, organism supplementary concept word, protocol supplementary concept word, rare disease supplementary concept word, unique identifier, synonyms, population supplementary concept word, anatomy supplementary concept word] #776009
4. 1 or 2 or 3 # 21456088
5. exp Nutrition Assessment/ 17699
6. exp Nutritional Status/ 53670
7. nutrition disorders/ or exp malnutrition/ or exp overnutrition/ 415551
8. exp Malnutrition/ 134754
9. exp Zinc/ or exp Anaemia, Iron-Deficiency/ or exp Trace Elements/ or exp Vitamins/ 719419
10. exp Obesity/ or exp Nutrients/ 845013
11. exp body mass index/ 148265
12. ("mid-upper arm circumference" or MUAC).mp. [mp=title, book title, abstract, original title, name of substance word, subject heading word, floating sub-heading word, keyword heading word, organism supplementary concept word, protocol supplementary concept word, rare disease supplementary concept word, unique identifier, synonyms, population supplementary concept word, anatomy supplementary concept word] 1834
13. BMI.mp. 190415
14. ("Nutritional status" or "nutrition assessment" or "malnutrition" or "undernutrition" or "overnutrition" or "micronutrient status" or "macronutrient status" or "BMI" or "MUAC" or "WHR" or CC or "arm span").mp. [mp=title, book title, abstract, original title, name of substance word, subject heading word, floating sub-heading word, keyword heading word, organism supplementary concept word, protocol supplementary concept word, rare disease supplementary concept word, unique identifier, synonyms, population supplementary concept word, anatomy supplementary concept word] 406089
15. 5 or 6 or 7 or 8 or 9 or 10 or 11 or 12 or 13 or 14 #1422182
16. "physical function".mp. 19460
17. exp "Activities of Daily Living"/ or exp "Quality of Life"/ 365043
18. exp muscle strength/ or exp hand strength/ 44338
19. exp physical functional performance/ 4054
20. exp Walking Speed/ 2654
21. ("Functional status" or "functional balance" or "functional capacity" or "functional ability" or "gait speed" or "physical activity" or "physical function" or "physical performance" or "mobility" or "muscle strength" or "handgrip strength" or "walking speed" or "short physical performance battery").mp. [mp=title, book title, abstract, original title, name of substance word, subject heading word, floating sub-heading word, keyword heading word, organism supplementary concept word, protocol supplementary concept word, rare disease supplementary concept word, unique identifier, synonyms, population supplementary concept word, anatomy supplementary concept word] 459975
22. 16 or 17 or 18 or 19 or 20 or 21 #793241
23. "sub-Sahara* Africa*".mp. 33791
24. ("Sub-Sahara* Africa" or Angola or Benin or Botswana or "Burkina Faso" or Burundi or Cameroon or "Cape Verde" or "Central African Republic" or Chad or Comoros or "Congo Democratic Republic" or "Congo Republic" or "Côte d'Ivoire" or Djibouti or Eritrea or Eswatini or Ethiopia or Gabon or Gambia or Ghana or Guinea or "Guinea Equatorial" or "Guinea-Bissau" or Kenya or Lesotho or Liberia or Madagascar or Malawi or Mali or Mauritania or Mauritius or Mayotte or Mozambique or Namibia or Niger or Nigeria or Rwanda or "São Tomé and Príncipe" or Senegal or Seychelles or "Sierra Leone" or Somalia or "South Africa" or Sudan or "South Sudan" or Tanzania or Togo or Uganda or Zambia or Zimbabwe).mp. [mp=title, book title, abstract, original title, name of substance word, subject heading word, floating sub-heading word, keyword heading word, organism supplementary concept word, protocol supplementary concept word, rare disease supplementary concept word, unique identifier, synonyms, population supplementary concept word, anatomy supplementary concept word] 496798
25. "africa south of the sahara"/ or exp africa, central/ or exp africa, eastern/ or exp africa, southern/ or exp africa, western/ 257282
26. 23 or 24 or 25 #515930
27. 4 and 15 and 22 and 26 #1184
